# Myeloid-associated differentiation marker is a novel SP-A-associated transmembrane protein whose expression on airway epithelial cells correlates with asthma severity

**DOI:** 10.1038/s41598-021-02869-w

**Published:** 2021-12-03

**Authors:** Alane Blythe C. Dy, Paul R. Langlais, Natalie K. Barker, Kenneth J. Addison, Sasipa Tanyaratsrisakul, Scott Boitano, Stephanie A. Christenson, Monica Kraft, Deborah Meyers, Eugene R. Bleecker, Xingnan Li, Julie G. Ledford

**Affiliations:** 1 Clinical Translational Sciences, University of Arizona Health Sciences, Tucson, AZ 85721 USA; 2 Asthma and Airway Disease Research Center, University of Arizona, Tucson, AZ 85724 USA; 3 Division of Endocrinology, Department of Medicine, University of Arizona, Tucson, AZ 85724 USA; 4 Department of Physiology, University of Arizona, Tucson, AZ 85724 USA; 5 Division of Pulmonary, Critical Care, Allergy and Sleep Medicine, Department of Medicine, University of California San Francisco, San Francisco, CA 94117 USA; 6 Department of Medicine, University of Arizona, Tucson, AZ 85724 USA; 7 Division of Genetics, Genomics and Precision Medicine, Department of Medicine, University of Arizona, Tucson, AZ USA; 8 Department of Cellular and Molecular Medicine, University of Arizona, Tucson, AZ 85724 USA; 9 1230 N Cherry Avenue, BSRL Building, Tucson, AZ 85719 USA

**Keywords:** Translational research, Asthma

## Abstract

Surfactant protein A (SP-A) is well-known for its protective role in pulmonary immunity. Previous studies from our group have shown that SP-A mediates eosinophil activities, including degranulation and apoptosis. In order to identify potential binding partners on eosinophils for SP-A, eosinophil lysates were subjected to SP-A pull-down and tandem mass spectrometry (MS/MS) analysis. We identified one membrane-bound protein, myeloid-associated differentiation marker (MYADM), as a candidate SP-A binding partner. Blocking MYADM on mouse and human eosinophils ex vivo prevented SP-A from inducing apoptosis; blocking MYADM in vivo led to increased persistence of eosinophilia and airway hyper-responsiveness in an ovalbumin (OVA) allergy model and increased airways resistance and mucus production in a house dust mite (HDM) asthma model. Examination of a subset of participants in the Severe Asthma Research Program (SARP) cohort revealed a significant association between epithelial expression of MYADM in asthma patients and parameters of airway inflammation, including: peripheral blood eosinophilia, exhaled nitric oxide (FeNO) and the number of exacerbations in the past 12 months. Taken together, our studies provide the first evidence of MYADM as a novel SP-A-associated protein that is necessary for SP-A to induce eosinophil apoptosis and we bring to light the potential importance of this previously unrecognized transmembrane protein in patients with asthma.

## Introduction

Asthma is a complex and heterogeneous disease affecting 339 million people worldwide^1^. It is one of the most common respiratory diseases, affecting both children and adults, characterized by airway inflammation, reversible airflow limitation and exacerbations. Eosinophils are a key immune cell present in 50–60% of asthmatics^2^ and their presence and dysregulation are clinically associated with more severe asthma^3^. Eosinophilia is defined as the presence^4^ of increased numbers of eosinophils in the airway and peripheral blood^5^, and is considered a major feature of type-2 high asthma^6–8^. The success of corticosteroid therapies and anti-eosinophil biologics, both of which contribute to the reduction of eosinophilia, albeit by different mechanisms, highlight the role eosinophils play in the pathology of asthma.

Surfactant protein A (SP-A) is produced mainly by type 2 alveolar cells but is also secreted in the conducting airways by club cells and cells in the submucosal glands^9,10^. There are two functional human SP-A genes, *SP-A1* and *SP-A2*, whose protein products come together to form an octadecamer^11^. In addition to its traditionally characterized effects on alveolar surface tension, this collectin structure provides a first-line of defense in pulmonary innate immunity by virtue of its ability to bind various cell surface receptors to trigger pro-inflammatory cascades, as well as, its ability to recognize and bind pathogen-associated molecular patterns for opsonization^12^. As such, SP-A can specifically bind to *Mycoplasma pneumoniae* (Mp), a pathogen associated with asthma exacerbations, to attenuate Mp pathogenicity and lung inflammation^13–16^.

SP-A is also known to modulate cytokines, IgE and eosinophil levels in the setting of type 2-associated allergic inflammation^17,18^ and treatment with exogenous SP-A in mouse models of asthma significantly reduces tissue eosinophilia^19^. We have determined that SP-A contributes to the resolution of eosinophilia by promoting eosinophil migration out of the lung tissue, and more remarkably, promoting eosinophil apoptosis in the lung lumen^20^. In addition to these phenotypes associated with resolution of eosinophilia, our group has also shown that SP-A inhibits eosinophil peroxidase release, a toxic product that can compromise the integrity of the airway epithelia^16^. Furthermore, a genetic variation in the carbohydrate recognition domain of SP-A2 occurring at position 223, which results in a glutamine (Q) to a lysine (K) substitution, alters regulatory functions of SP-A in apoptosis and degranulation of eosinophils^11,20^.

While the regulation of eosinophil activities by SP-A is evident, the precise mechanisms are unknown. This current study sought to determine components of the eosinophil with which SP-A interacts with to mediate eosinophil apoptosis. First, we identified a putative eosinophil-associated protein binding partner for SP-A, Myeloid-Associated Differentiation Marker (MYADM) using quantitative proteomics. We then show a functional role for MYADM in: (1) SP-A-mediated eosinophil apoptosis of murine and human eosinophils in vitro and (2) resolution of eosinophilia and airway hyperresponsiveness after allergen challenge in vivo. Finally, using a subset of participants in the Severe Asthma Research Program (SARP) cohort, we are the first to describe a significant association between epithelial expression of MYADM and parameters of airway inflammation, including peripheral blood eosinophilia. Taken together, our studies show that MYADM is a novel association partner for SP-A on eosinophils that is necessary for SP-A to induce eosinophil apoptosis and we bring to light the potential importance of this previously unrecognized transmembrane receptor in patients with asthma.

## Materials and methods

### Human SP-A extraction

SP-A was extracted from the lung lavage of alveolar proteinosis patients as previously described^16,21^. Briefly, using butanol extraction methods, SP-A was isolated, subsequently pelleted and solubilized in octylglucoside and 5 mM Tris at pH 7.4. SP-A was then passed through a polymyxin B-agarose column for endotoxin removal. Final endotoxin levels were less than 0.01 pg/mg of extracted SP-A (Pierce LAL Chromogenic Endotoxin Quantitation Kit, ThermoFisher Scientific, Waltham, MA).

### Eosinophil and leukocyte isolation

#### Murine eosinophils

Eosinophils from the blood of IL-5 transgenic mice were isolated and purified as previously described^16,20^. Briefly, blood was collected by cardiac puncture through the left ventricle, after which red blood cells were lysed and eosinophils isolated by negative selection. Cytospin preparations stained with the Easy III rapid differential staining kit (Azer Scientific, Morgantown, PA) was used to check purity and was verified to be greater than 95%.

#### Human leukocytes

Leukocytes were isolated from the blood of asthmatic volunteers as previously described^20^. Volunteers consisted of six non-Hispanic, white patients, one male and five females, between the ages of 21 and 59, and were either on Albuterol only or on combination Albuterol/Symbicort, Albuterol/Ipratropium, Albuterol/Advair therapies except for one patient not on medication. Density gradient centrifugation was performed using 37% Optiprep (Alere Technologies AS, Oslo, Norway) and centrifuged at 1500 RCF for 30 min at 20 °C and no brake. Red blood cells were subsequently lysed and resulting pellet resuspended in RPMI 1640 (10% FBS). All human studies were conducted on protocols approved by the Institutional Review Board (IRB) at the University of Arizona under Principle Investigator, Dr. Ledford. All methods were carried out in accordance with relevant guidelines and regulations. Informed consent was obtained from all subjects.

### Pull-down, in-gel digestion and liquid chromatography tandem mass spectrometry (LC–MS/MS)

#### Pull-down

For each pull-down, mouse eosinophil cell lysates (~ 3 mg) were incubated with SP-A (5 μg) conjugated to protein A sepharose beads (Sigma, St. Louis, MO) overnight on a tube rotator at 4 °C. The pull-downs were washed with cold PBS and eluted twice at 95 °C using 8 μl of SDS sample loading buffer (4% SDS, 0.067 M Tris, 8.0 M urea, 0.04% bromophenol blue, 5% β-mercaptoethanol) for 4 min each time. The resulting eluate was loaded onto Mini-Protean TGX precast gels (10% SDS-PAGE, Bio-Rad Laboratories, Hercules, CA) and stained with Bio-safe Coomassie G-250 stain (Bio-Rad Laboratories, Hercules, CA).

#### In-gel digestion

Each lane of the SDS-PAGE was cut into five slices and processed similarly to previously described methods^22,23^. Each slice was destained twice using 50% acetonitrile (ACN) and 100 mM NH_4_HCO_3_. Slices were subsequently dehydrated with 100% ACN at room temperature for 15 min, after which the ACN was removed by aspiration and samples were dried by vacuum centrifugation for 30 min at 62 °C. Trypsin (0.01 μg/μl, 100 mM NH_4_HCO_3_) was added to the gel pieces for 15 min at 4 °C before addition of 100–150 μl of NH_4_HCO_3_, ensuring that the level of liquid is 3 mm above the gel pieces. Samples were digested overnight (maximum of 16 h) with continuous shaking at 300 rpm at 37 °C. Extraction of peptides from the gel pieces was performed using 5% formic acid (FA) and dried down to ~ 10 μl by vacuum centrifugation at 62 °C. Ten microliters of 0.1% heptafluorobutyric acid (HFBA)/4% FA (v/v) was added to this dried down extract and incubated for 15 min at room temperature. Samples were then loaded onto a solid-phase Millipore ZipTip C18 (Sigma, St. Louis, MO), first washed with 0.005% HFBA/5% FA (v/v), then eluted with 1% FA/50% ACN (v/v), followed by 1% FA/80% ACN (v/v). The resulting eluates were dried by vacuum centrifugation for 25 min at 62 °C.

#### Mass spectrometry

HPLC–MS/MS was performed on an Orbitrap Fusion Lumos Tribrid mass spectrometer (ThermoFisher Scientific, Waltham, MA) coupled to an UltiMate 3000 RSLCnano UHPLC system (ThermoFisher Scientific) with an EASY-Spray C18 liquid chromatography column (ThermoFisher Scientific) as previously described^23,24^. Resulting mass spectra were searched against SwissProt database using Mascot (Matrix Science, Boston, MA). Spectral abundance counts were quantified and validated with Scaffold (Proteome Software, Portland, OR).

### Assessment of eosinophil viability

#### xCELLigence Real-time Cell Analyzer

Real-time monitoring of eosinophil cytotoxicity was performed using an xCELLigence Real-Time Cell Analyzer (ACEA Biosciences, San Diego, CA), measuring electrical impedance as previously described^20^. Briefly, after a background reading with media only, eosinophils (1 × 10^6^ cells per well), pre-treated with vehicle or an anti-MYADM (Invitrogen, Carlsbad, CA), were seeded onto 96-well plates with fused gold microelectrode biosensors on the bottom surface and allowed to equilibrate for ~ 5 h. An initial dose response curve for anti-MYADM was carried out including 0, 3, 10, 30 μg/ml per 1 × 10^6^ eosinophils, after which it was determined that 1 μg/ml was optimal to block the action of SP-A per ~ 33,000 eosinophils. SP-A was then added at various concentrations and changes in electrical impedance were observed for 24 h. In order to compare results from individual wells, changes in electrical impedance are presented as “normalized cell index” values as previously described^25,26^. Area under the curve (AUC) measurements were derived from plots of normalized cell index values over time to quantify eosinophil cytotoxicity.

#### Flow cytometry analysis

Isolated human leukocytes were pre-treated with anti-MYADM (Invitrogen PA1-41578, Carlsbad, CA) that interacts with both human and mouse or vehicle and incubated with SP-A overnight (16–17 h). Cells were then washed, resuspended in FACS buffer (2% FBS in PBS) and labeled with anti-Siglec-8-PE (BioLegend, San Diego, CA). Apoptotic cells were labeled with a FITC Annexin V Apoptosis Detection Kit (BD Biosciences, San Diego, CA). Eosinophils were assessed for apoptosis using an Attune NXT Flow Cytometer (ThermoFisher Scientific, Waltham, MA) and identified as the following: Live (Siglec-8^+^, Annexin V^−^, PI^−^), Early apoptotic (Siglec-8^+^, Annexin V^+^, PI^−^) and Late apoptotic/dead (Siglec-8^+^, Annexin V^+^, PI^+^). Data were processed and analyzed using FlowJo 10.5.3.

### Human subjects

A subset of participants (n = 156) from the Severe Asthma Research Program (SARP) cohort III, funded by NHLBI, with mild to severe asthma and healthy controls were included in this analysis^27–29^. Bronchial brush biopsies were collected for RNA-seq analysis. In brief, Illumina HiSeq RNAseq reads were mapped to human genome hg38 using STAR package^30^. Read counts were regularized logarithm transformed using DESeq2 package^31^. Airway epithelial cell (AEC) mRNA expression levels of MYADM were natural logarithm transformed into normally distributed variable. Then, MYADM mRNA levels were examined for correlation with asthma status and other parameters related to asthma, namely, baseline FEV_1_, baseline FEV_1_/FVC, blood and sputum eosinophil counts, FeNO, total serum IgE levels and number of exacerbations in the last 12 months, using a generalized linear model, adjusting for age, sex, race, body mass index (BMI) and group effect^29^.

### Mouse models

All animal experiments were performed in accordance with protocols approved by the Institutional Animal Care and Use Committee (IACUC) at the University of Arizona under protocol number 15-575 (Dr. Ledford). All experiments were performed in accordance with relevant guidelines and regulations and adhere to ARRIVE guidelines for the reporting of animal experiments. IL-5 transgenic mice on a C57BL/6 background were generously provided by the late Dr. James Lee^32^ and bred in-house for in vitro eosinophil studies. Wild-type C57BL/6 mice (Jackson Laboratories, Bar Harbor, ME), 8 weeks of age at the beginning of the studies, were used for both the house dust mite (HDM) and ovalbumin (OVA) experimental allergen challenges. All animals were housed under 12-h dark/light cycles with free access to standard chow and water in a conventional animal facility.

#### Induction of allergic airways inflammation with OVA

Mice were first sensitized using OVA (50 μg in alum) or sterile saline intraperitoneally (i.p.) on days 0 and 7 and subsequently challenged intranasally (i.n.) on days 14, 15 and 16. One day after the last challenge, mice were given a MYADM antibody detailed above (Oropharyngeal, 0.1 mg/kg body weight) or sterile saline. Mice were assessed on days 18 and 22.

#### Induction of allergic airways inflammation with HDM

HDM (100 μg in 50 μl per mouse) or sterile saline was given intranasally (i.n.) on days 0, 7 and 14. One day after the last challenge, mice were given a MYADM antibody (Oropharyngeal, 0.1 mg/kg body weight) or sterile saline. Mice were assessed on day 20.

### Bronchoalveolar lavage

On days 18 and 22 of OVA challenge, mice were euthanized by anesthetic overdose (urethane, 250 mg/ml, 1.5 g/kg; Sigma Aldrich, St. Louis, MO). The trachea was then exposed and cannulated with a 19G catheter. Airways and lungs were lavaged with 1.5 ml PBS (100 μM EDTA). Total cell counts were quantified using a Countess II FL Automated Cell Counter (Life Technologies, Carslbad, CA). Cytocentrifuged slides at a seeding density of ~ 200,000 cells per slide were stained with the Easy III Rapid Differential Staining Kit (Azer Scientific, Morgantown, PA). Differential leukocyte counts were assessed by standard morphological identification of cell types.

### Pulmonary function studies by FlexiVent

For mouse models of experimental allergen challenge, mice were anesthetized with urethane (125 mg/ml, Sigma, St. Louis, MO) by intraperitoneal (i.p.) injection at 16 μl/g body weight, followed by pancuronium bromide (0.8 mg/ml in saline, Sigma, St. Louis, MO) by i.p. injection at 10 μl/g body weight. The experimental treatment groups of mice were blinded to the person operating the Flexivent machine. The trachea was then exposed and cannulated with a 19G catheter. Using a computer-controlled piston-ventilator (FlexiVent, SCIREQ Inc., Montreal, Quebec, Canada), mice were subsequently ventilated with a tidal volume of 10 ml/kg body weight, a frequency of 150 breaths/min and a positive end-expiratory pressure of 3 cm H_2_O. Two deep inflations were successively applied to open closed lung areas and standardize lung volume. Single-frequency (Snapshot-150) and broadband (Quick Prime-3) forced oscillation technique (FOT) measurements were performed to measure total (R_rs_) and Newtonian airway resistance (R_n_), respectively. Only male mice were used for Flexivent analysis on lung function as female mice are very low responders on this system.

#### OVA-challenged mice

On day 18 of OVA challenge, mice were examined for acute responses to methacholine challenge as previously described^33^. Briefly, single-frequency and broadband forced oscillation technique measurements were repeatedly alternated within a few seconds of each other for approximately three minutes to obtain a total of 12 measurements for each methacholine dose. This protocol was performed for increasing concentrations of methacholine (0, 3, 10, 30, 100 mg/ml). Total airway resistance (R_rs_) and elastance (E_rs_) of the respiratory system were quantified from single-frequency FOT, while Newtonian airway resistance (R_n_) was quantified from broadband FOT measurements.

#### HDM-challenged mice

Baseline, unchallenged (no methacholine) pulmonary mechanics were measured on day 20 of HDM challenge. An additional set of mice was examined on day 15 of the HDM model in order to compare lung function at the peak of inflammation (day 15) to the resolution phase (day 20). One single-frequency and one broadband FOT measurement were performed to assess baseline differences in total and Newtonian airway resistance, respectively.

### Gene expression by real-time quantitative RT-PCR

To evaluate mRNA expression of GAPDH (internal control; sense: 5′-CCT GCA CCA CCA ACT GCT TA-3′, antisense: 5′-GTC TTC TGG GTG GCA GTG AT-3′) and Muc5ac (sense: 5′-GAG GGC CCA GTG AGC ATC TCC-3′, antisense: 5′-TGG GAC AGC AGC AGT ATT CAG T-3′), real-time quantitative RT-PCR was performed. Airways and lungs of each mouse were washed with 1.5 ml of PBS (100 μM EDTA). The right lung lobes were removed, snap frozen in liquid nitrogen and stored at − 80 °C until assayed. Total lung RNA was extracted by TRIzol (Invitrogen, San Diego, CA) and subsequently reverse transcribed using an iScript cDNA synthesis kit (Bio-Rad Laboratories, Hercules, CA). Targets were then amplified using the respective primers listed above and SYBR Green Supermix with the Bio-Rad CFX system (Bio-Rad Laboratories, Hercules, CA). After standardization to the internal control, relative cycle threshold values (CT) using the 2^−ΔΔCT^ equation were calculated to compare gene expression levels and presented as fold change over saline-treated control^34^.

### Histological analysis

Left lung lobes from each mouse were fixed in 10% formalin upon necropsy and subsequently embedded in paraffin. Four microns-thick midsagittal lung sections were stained with Periodic Acid Schiff stain and were scored by blinded individuals at 10 × magnification. Scores from individual histological slides were averaged within each treatment group for statistical analysis.

### Statistical analysis

Statistical analyses of murine experiments were done using GraphPad Prism software. Multiple groups were assessed for global differences using one-way ANOVA, followed by multiple *t* tests with Bonferonni’s correction for multiple comparisons. Two-way ANOVA was used for analysis of data from methacholine challenge to examine the influence of both methacholine dosage and treatment, followed by multiple *t* tests.

Clinical characteristics of participants from SARP III cohort were presented as percentages for categorical variables, means with standard deviations for normally distributed continuous variables, and medians with first and third quartiles for continuous variables not in normal distribution. Three-group comparisons (healthy control vs. non-severe asthma vs. severe asthma) were performed using Kruskal–Wallis test for continuous variables or chi-square test for categorical variables.

## Results

### SP-A pulls-down membrane-associated protein myeloid-associated differentiation marker (MYADM)

**Figure 1:**
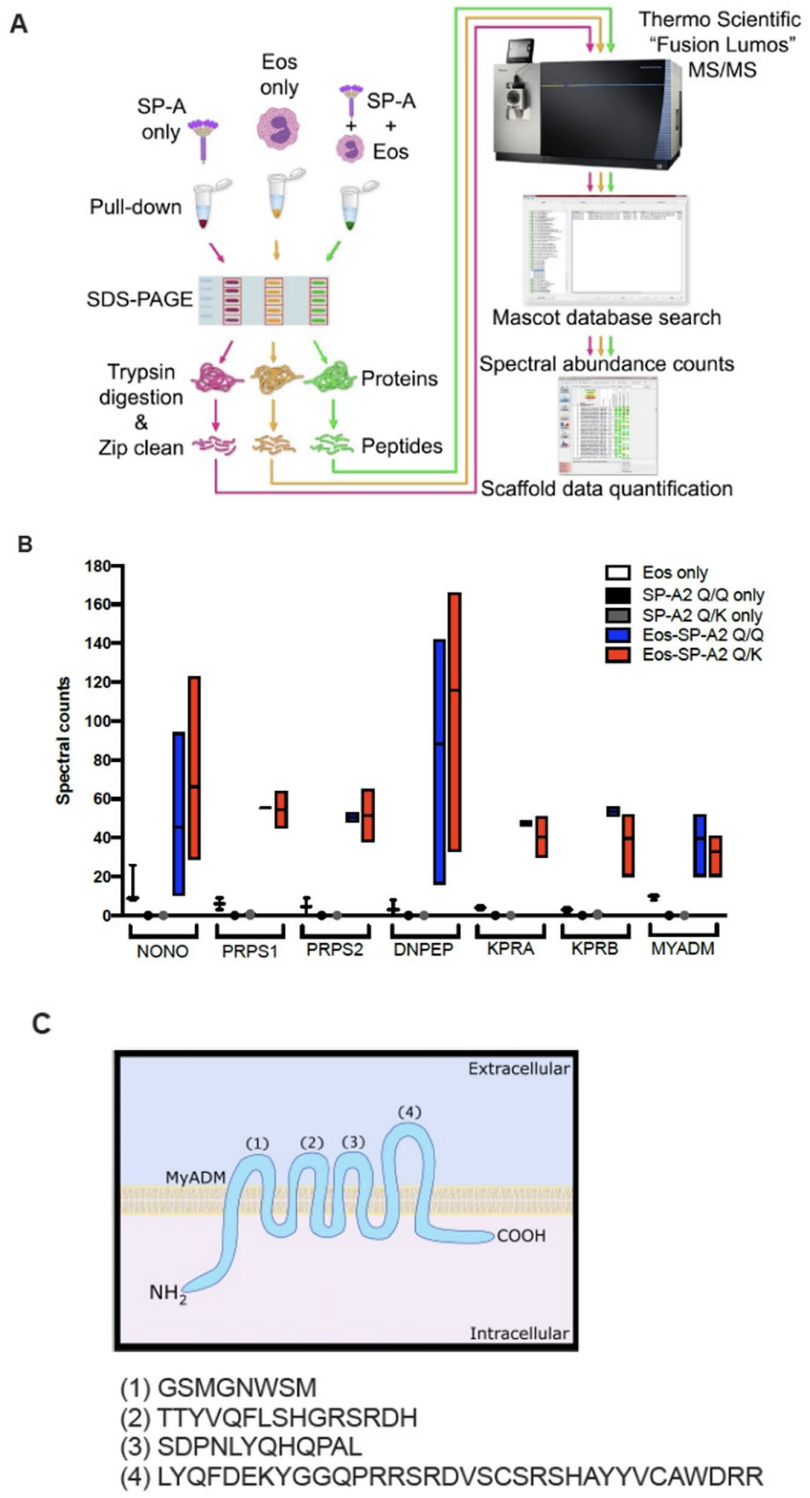
Identification of eosinophil-associated proteins interacting with SP-A. (**A**) Schematic of the experimental design for the quantitative proteomics approach by mass spectrometry to identify interacting proteins between SP-A and eosinophils. (**B**) Spectral counts of eosinophil-associated proteins immunoprecipitated with SP-A by LC–MS/MS. Data are presented as the mean ± SEM and are from three independent experiments. (**C**) Schematic of the extracellular, transmembrane and intracellular domains of the multi-pass membrane associated protein, myeloid-associated differentiation marker (MYADM). (1), (2), (3), (4) = amino acid sequences of the extracellular domains of MYADM.

To identify potential SP-A interacting protein partners on the eosinophil, we performed pull-down assays on eosinophil lysates with SP-A and analyzed the resulting eluates by liquid chromatography tandem mass spectrometry (LC–MS/MS) (Fig. 1A). Purified SP-A preparations that were genotyped as homozygous for Q (Q/Q) or heterozygous (Q/K) at position 223 were used to discern potential differences in interaction due to genetic variation (Fig. 1A). Seven proteins, listed in Table 1, were enriched in the SP-A:eosinophil preparation over controls independent of the genetic variation at position 223 (Table 1, Fig. 1B). NONO, PRPS1, PRPS2, DNPEP, KPRA and KPRB are intracellular proteins, whereas MYADM is membrane-associated^35^. MYADM is a multi-pass membrane protein that has 8 membrane spanning regions (Fig. 1C), whose most well-known function is influencing differentiation of myeloid cells^35,36^. Since our primary objective was to identify potential targets for extracellular binding of SP-A to eosinophils and MYADM was the sole membrane-associated protein from Fig. 1, this was the best candidate from our analyses to investigate further.

**Table 1:** Eosinophil-associated proteins immunoprecipitated with SP-A by liquid chromatography tandem mass spectrometry (LC–MS/MS). Table reports spectral counts (SE) from LC–MS/MS analyses of eosinophil-associated proteins immunoprecipitated with SP-A.

Protein name		Eosinophils only (SE)	SP-A2 Q/Q only (SE)	SP-A2 Q/K only (SE)	Eos-SP-A2 Q/Q (SE)	Eos-SP-A2 Q/K (SE)
Non-POU domain-containing octamer-binding protein	NONO	14.33 (5.84)	0	0	45.33 (25.15)	66.33 (28.81)
Ribose phosphate pyrophosphokinase 1	PRPS1	6.00 (3.00)	0	0	55.50 (0.50)	54.50 (9.50)
Ribose phosphate pyrophosphokinase 2	PRPS2	4.50 (4.50)	0	0	50.50 (2.50)	51.50 (13.50)
Aspartyl aminopeptidase	DNPEP	3.67 (2.33)	0	0	88.00 (37.47)	115.67 (41.66)
Phosphoribosyl pyrophosphate synthase-associated protein 1	KPRA	4.00 (1.00)	0	0	47.50 (1.50)	40.50 (10.50)
Phosphoribosyl pyrophosphate synthase-associated protein 2	KPRB	3.00 (1.00)	0	0.50 (0.50)	53.50 (2.50)	50.50 (12.50)
Myeloid-associated differentiation marker	MYADM	9.67 (0.88)	0	0	39.67 (9.94)	33.00 (6.56)

### MYADM blockade abrogates the cytotoxic effect of SP-A on eosinophils in vitro

**Figure 2:**
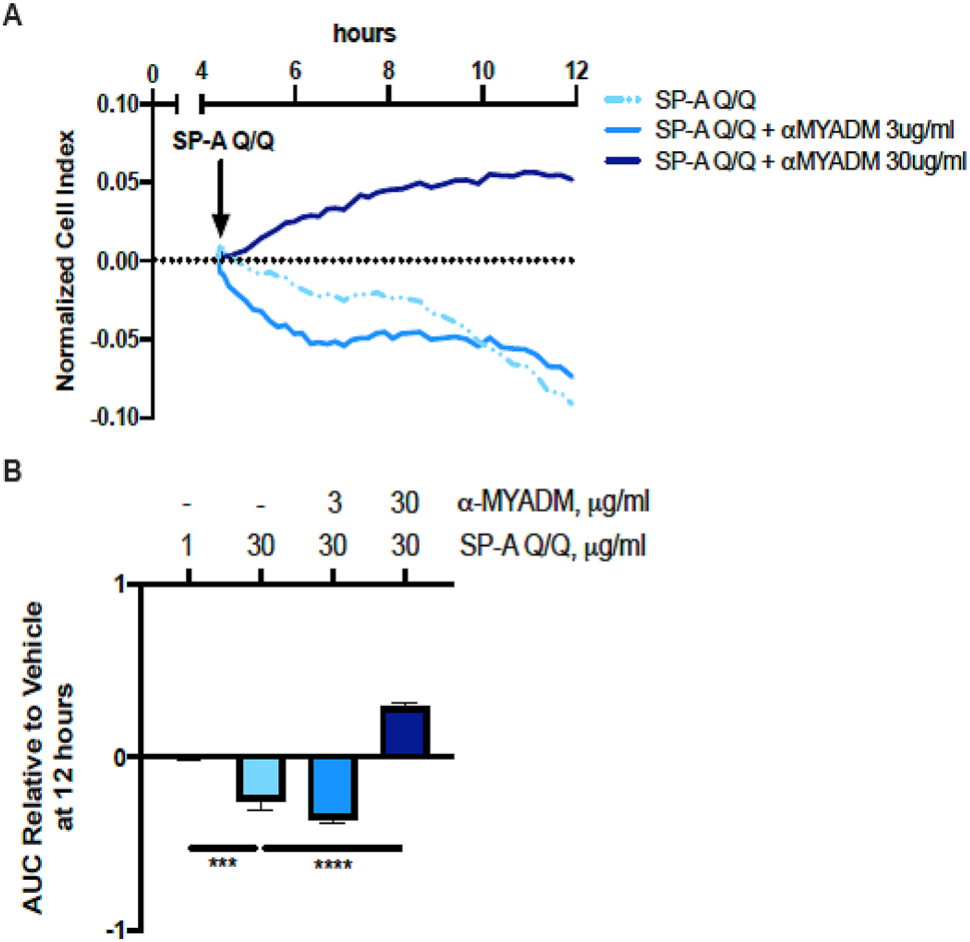
Evaluation of the role of MYADM in the cytotoxic effect of SP-A on mouse eosinophils in vitro. (**A**) RTCA tracings of normalized cell indices and (**B**) calculated area under the curve (AUCs) of the dose response from the in vitro stimulation of mouse eosinophils by SP-A (30 μg/ml) with or without antibody to block MYADM. One-way ANOVA with Bonferonni’s correction for multiple comparisons. ****p < 0.0001, n = 3 replicates per condition.

Our next question was whether MYADM was involved in pro-apoptotic events initiated by SP-A. To assess whether MYADM is indeed involved in the cytotoxic effect of SP-A on eosinophils, we incubated purified eosinophils from the peripheral blood of IL-5 transgenic mice with SP-A in the presence or absence of MYADM antibody. As previously shown^20^, there was a decrease in the normalized cell index of eosinophils using xCELLigence Real-Time Cell Analyzer (RTCA) upon addition of SP-A as a result of its apoptotic effect (Fig. 2A). However, eosinophils pre-treated with anti-MYADM did not have the same change in normalized cell index (Fig. 2A). In line with this, there was a statistically significant decrease in the calculated area under the curve (AUC) in eosinophils incubated with SP-A, which was absent with anti-MYADM pre-treatment (Fig. 2B).

**Figure 3:**
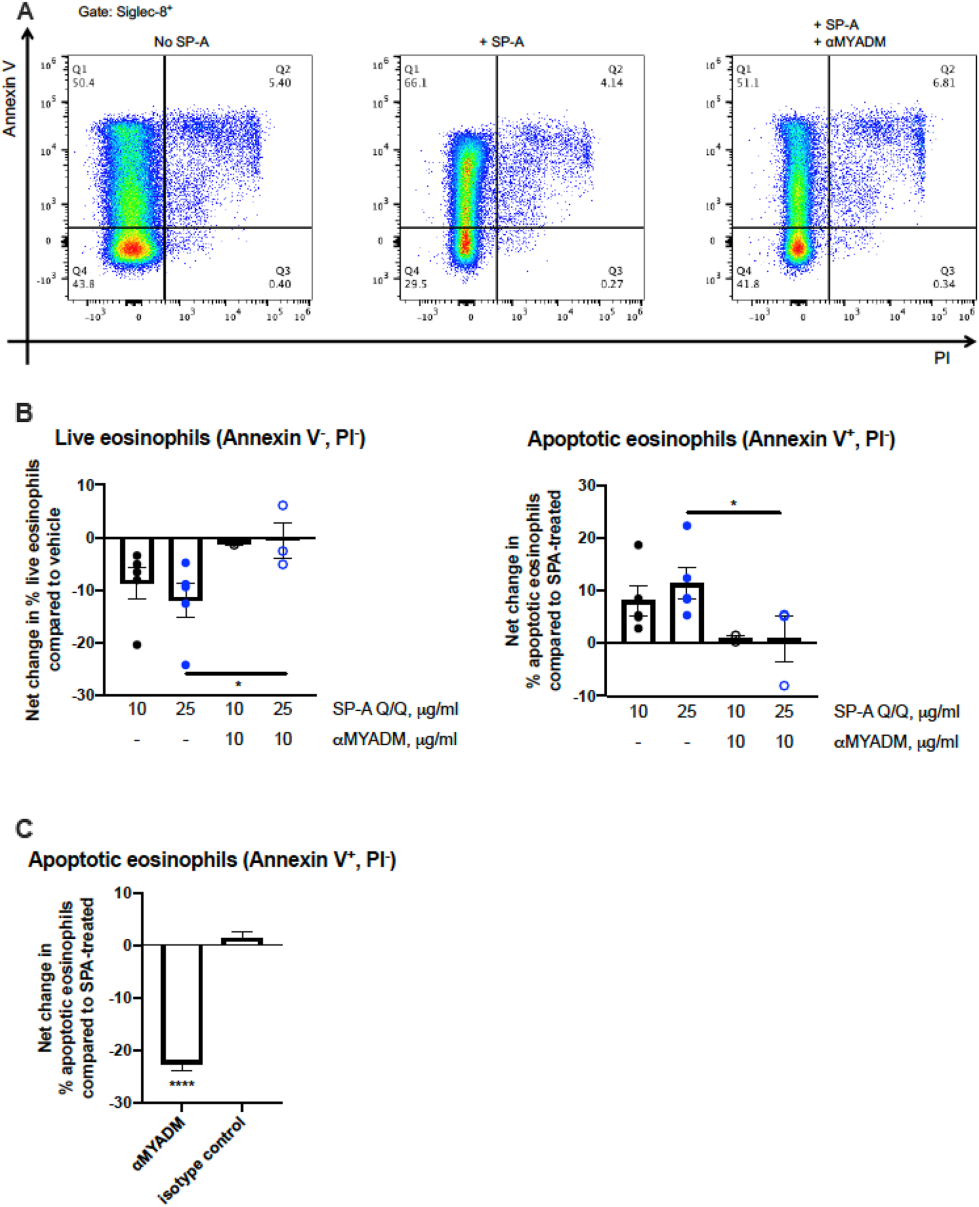
Evaluation of the role of MYADM in the cytotoxic effect of SP-A on human eosinophils in vitro. (**A**) Representative flow diagrams of human eosinophil apoptosis and cell death by Annexin V and PI after 16 h incubation with SP-A after direct treatment with SP-A. live = Annexin V^−^, PI^−^, early apoptosis = Annexin V^+^, PI^−^, late apoptosis/dead = Annexin V^+^, PI^+^. (**B**) Quantification of percent (%) change in live and apoptotic eosinophils from vehicle control. (**C**) Percent (%) change in apoptotic eosinophils from SP-A-treated. One-way ANOVA with Bonferroni’s correction for multiple comparisons or unpaired *t* test, *p < 0.05, ****p < 0.0001. Data (mean ± SEM) are from three independent experiments with n = 2–3 replicates per condition.

To determine whether human eosinophils isolated from asthma patients would respond similarly, we isolated leukocytes from the peripheral blood and analyzed these cells by flow cytometry after incubation with SP-A, with or without pre-treatment with anti-MYADM. Due to the low quantity of eosinophils recovered from asthma patients, these studies were analyzed by flow cytometry as opposed to RTCA analysis. Staining with Annexin V and PI showed that the shift from live (Annexin V^−^, PI^−^) to early apoptotic (Annexin V^+^, PI^−^) was diminished in eosinophils (Siglec-8^+^) incubated with SP-A when anti-MYADM was present compared to eosinophils incubated with SP-A alone (Fig. 3A). Quantification of the differences in live and apoptotic cells between eosinophils incubated with vehicle and (1) eosinophils incubated with SP-A only or (2) eosinophils incubated with SP-A in the presence of anti-MYADM confirm that pre-treatment with anti-MYADM reduces the ability of SP-A to promote eosinophil apoptosis in human eosinophils as well (Fig. 3B). Whereas we observed a robust mean decrease of 22.5% (SEM = 1.24) in apoptotic eosinophils with pre-treatment using anti-MYADM when compared to eosinophils treated with SP-A only, an isotype control in place of anti-MYADM showed a negligible mean increase of 1.42% (SEM = 1.24) in apoptotic eosinophil counts when compared to eosinophils treated with SP-A only (Fig. 3C). This confirmed that SP-A was not being depleted as a result of non-specific binding to the antibodies used.

### Disruption of the SP-A:MYADM interaction leads to a delay in resolution of airway eosinophilia after OVA allergen-challenged

In Figs. 2 and 3, we provide evidence that SP-A is unable to effectively promote eosinophil apoptosis when MYADM was blocked in functional assays in vitro. Since eosinophils are well-known contributors to type 2 high asthma^6^ and their accumulation in the airways are significantly associated with asthma severity^3,37,38^, we sought to determine whether the SP-A:MYADM interaction was important for eosinophilia in vivo.

**Figure 4:**
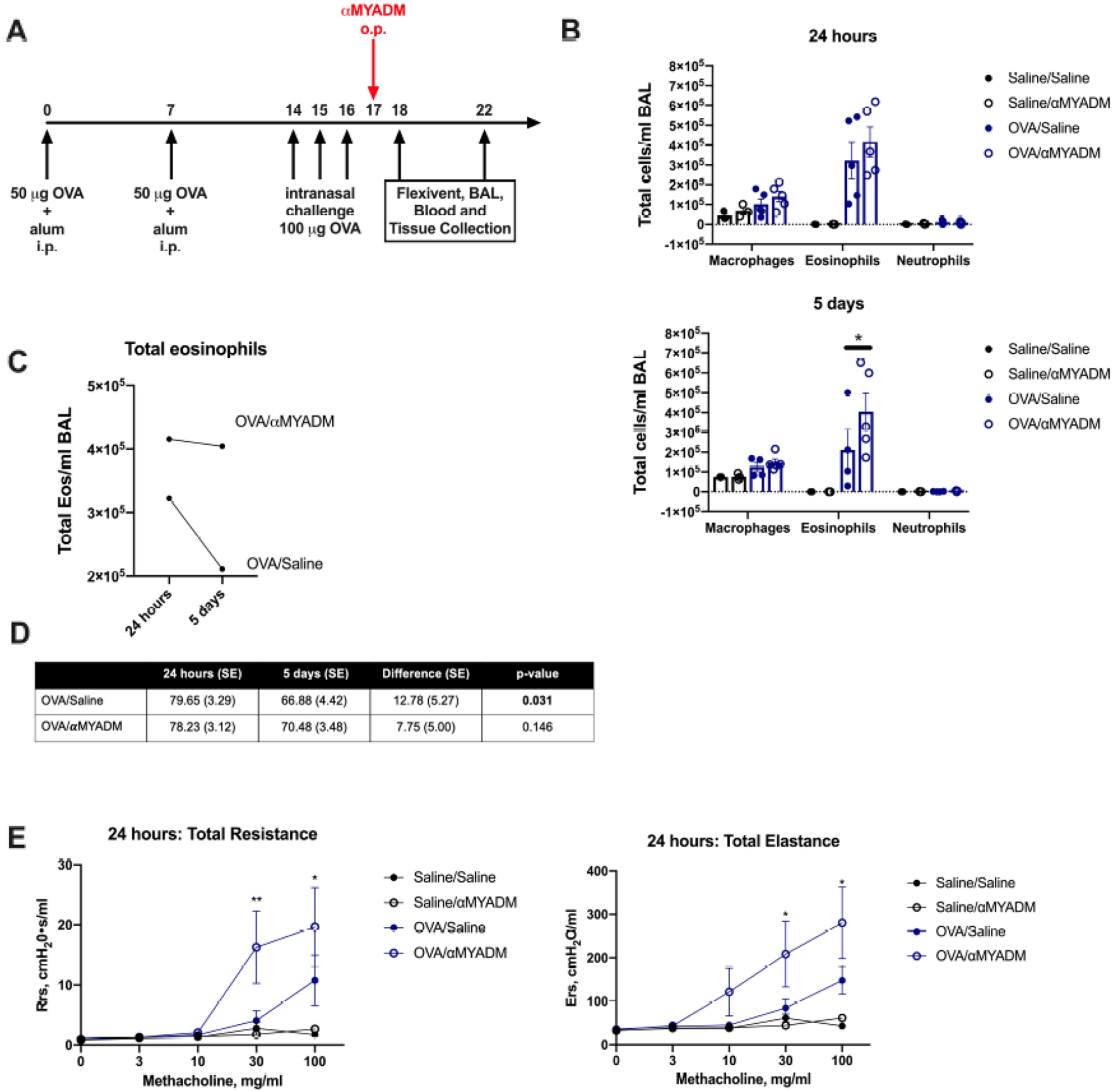
Evaluation of the role of MYADM on eosinophilia and airway reactivity to methacholine challenge using an in vivo Ova model. (**A**) Schematic of experimental allergen challenge by Ova, with or without antibody to MYADM. Quantification of (**B**) all cell types, (**C**) total eosinophils and (**D**) differences in means of percent (%) eosinophils in the BAL 24 h and 5 days after anti-MYADM administration. (**E**) Total resistance (R_rs_) and total elastance (E_rs_) after methacholine challenge at 24 h after anti-MYADM administration. One-way or two-way ANOVA with multiple *t* tests, *p < 0.05, **p < 0.01. Data (mean ± SEM) are from n = 4–5 mice per treatment group.

To determine whether disruption of the SP-A:MYADM interaction would affect the resolution of eosinophilia after in vivo experimental allergen challenge, wild-type mice were challenged with OVA or sterile saline and were given anti-MYADM via oropharyngeal delivery one day after terminal challenge (Fig. 4A). At 24 h after administration of anti-MYADM, quantification of cells in the bronchoalveolar lavage (BAL) revealed a robust recruitment of eosinophils in both OVA-challenged mice, with those which received anti-MYADM (OVA/αMYADM mice) having a slightly higher mean total eosinophils (Fig. 4B,C). However, when comparing total eosinophils 5 days post anti-MYADM administration, OVA-challenged mice that received sterile saline (OVA/saline mice) had significantly less eosinophils in the BAL compared to OVA/αMYADM mice (Fig. 4B,C). In fact, both total eosinophils per milliliter of BAL and percent eosinophils when plotted across the two time points show a distinct difference in the slope of the lines between the two OVA-challenged groups, with OVA/saline mice having a significantly steeper decline in eosinophils compared to OVA/αMYADM mice, which had more pronounced persistence in eosinophilia (Fig. 4C,D).

### Disruption of SP-A:MYADM interaction leads to increased airway resistance in OVA-challenged and HDM-challenged mice

In addition to a delay in the resolution of eosinophilia, OVA/αMYADM mice had the highest responses to methacholine challenge as assessed by pulmonary function measurements via FlexiVent on day 18 (Fig. 4E). Specifically, compared to OVA/saline, OVA/αMYADM mice had significantly increased total resistance (R_rs_) and total elastance (E_rs_) (Fig. 4E). Notably, at a dose of 100 μg/ml of methacholine, the R_n_ values of OVA/αMYADM were persistently elevated from days 18 to 22, whereas a downtrend in the central airways (Newtonian) resistance can be observed in the OVA/saline mice (Supp. Fig. 1).

**Figure 5:**
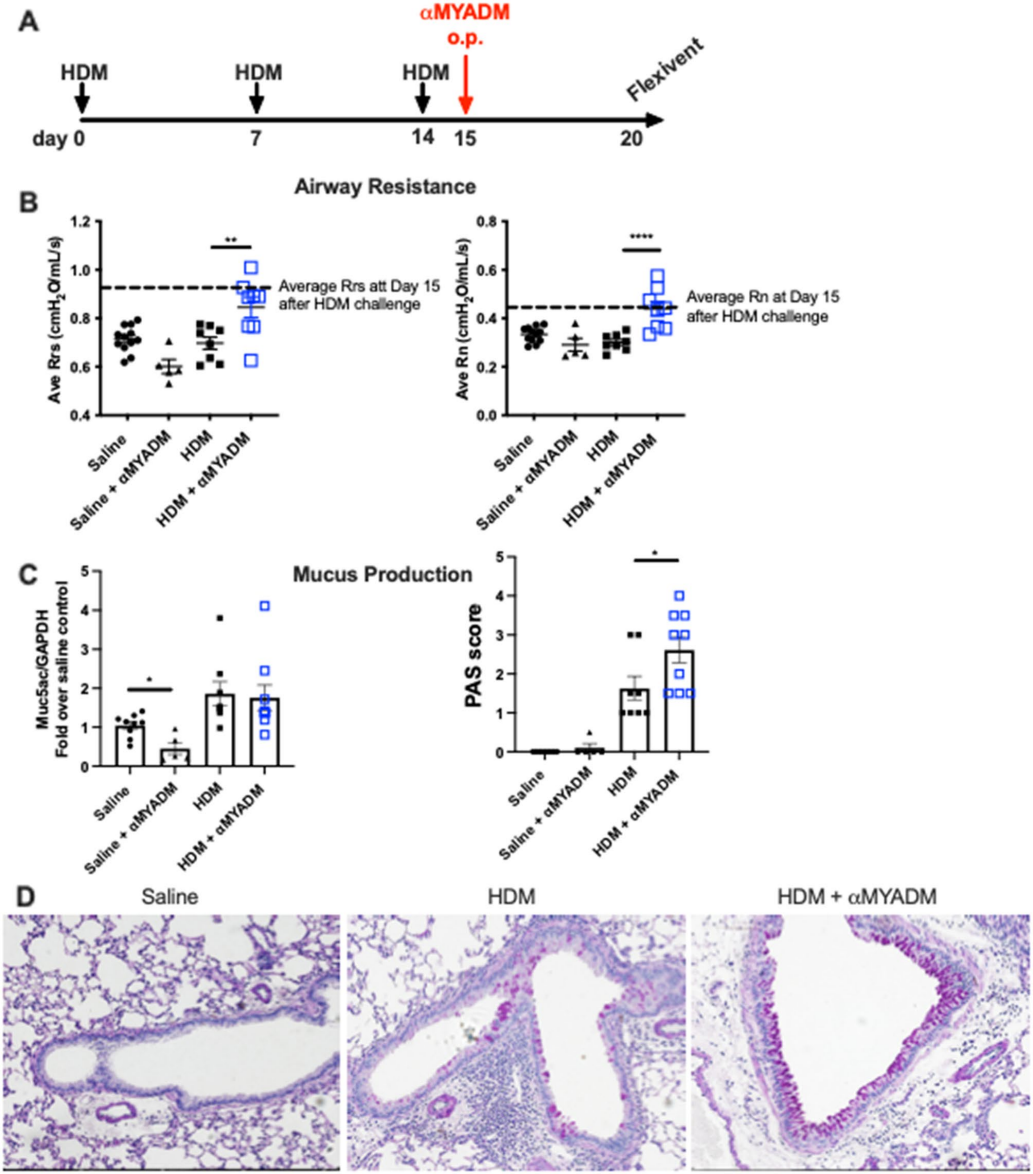
Evaluation of the role of MYADM in lung function using an in vivo HDM model. (**A**) Schematic of experimental allergen challenge by HDM, with or without antibody to MYADM. (**B**) Total airway resistance (Rrs, left panel) and Newtonian resistance (Rn, right panel) of wild-type mice 6 days after terminal HDM challenge. No methacholine was used for these measurements. Dotted line: average resistance of HDM-challenged mice 1 day after terminal HDM challenge. (**C**) Muc5ac gene expression (left panel) and PAS Scoring (right panel) of lungs from wild-type mice 6 days after terminal HDM challenge. One-way ANOVA with Bonferroni’s correction for multiple comparisons, **p < 0.01, ****p < 0.0001. Data (mean ± SEM) are from two independent experiments with n = 4–5 mice per treatment group. (**D**) Representative PAS stained sections taken at 20X magnification.

Studies were also conducted using the less eosinophilic, but more relevant environmental allergen, HDM model to assess the importance of the SP-A:MYADM interaction to asthma in vivo. Wild-type mice were challenged with HDM or sterile saline and were given anti-MYADM via oropharyngeal delivery one day after terminal challenge (Fig. 5A). Assessment of baseline (no methacholine) pulmonary function measurements were performed to quantify the degree of total airway resistance (R_rs_) and the resistance of the conducting airways (R_n_, Newtonian resistance) six days after terminal challenge. HDM-challenged mice that received anti-MYADM (HDM/αMYADM) had significantly increased total (Fig. 5B, left panel) and Newtonian resistance (Fig. 5B, right panel) compared to HDM-challenged mice that received sterile saline (HDM/saline). Moreover, HDM/αMYADM mice had airway resistance values that were more similar to the average resistance of HDM-challenged mice one day after terminal challenge (Fig. 5B, dotted line).

To investigate potential factors contributing to the increased airway resistance observed in HDM/αMYADM mice, mucus production was evaluated by assessing total lung Muc5ac gene expression and Periodic Acid Schiff (PAS)-stained lung sections (Fig. 5C,D). While Muc5ac gene expression was not different between HDM/saline mice and HDM/αMYADM mice at six days post-terminal challenge, mice given anti-MYADM exhibited increased mucus as assessed by PAS scoring in lung tissue (Fig. 5C,D). Surprisingly, saline-treated mice that were administered a single dose of MYADM blocking antibody had significantly lower Muc5ac gene expression compared to saline only control mice (Fig. 5C, left panel). Since protein levels were low to non-detectable in the non-allergic mice, it is likely the impact of gene expression corresponding to protein levels is non evident in an unchallenged state.

### MYADM gene expression is associated with asthma severity and type 2 inflammation phenotypes

To determine if MYADM expression had any relevance to human asthma, a subset of the Severe Asthma Research Program (SARP) III (Table 2), a longitudinal cohort as previously described^28,29^, was analyzed using a generalized linear model, adjusting for baseline characteristics. Data was available for n = 156 individual participants, of which n = 42 healthy controls, n = 49 non-severe asthma, n = 65 severe asthma. Overall, the non-severe asthma participants were younger than the other two groups and the healthy controls had lower BMI as compared to the asthma groups. As expected, FEV_1_ (% predicted) and FEV_1_/FVC ratios were significantly lower in the asthma groups as compared to healthy controls. There was no difference in the ethnicities of participants across the three groups.

**Table 2:** Demographics of SARP3 patient samples. Participant samples from the SARP3 dataset were assessed in our study: n = 42 healthy controls, n = 49 non-severe asthma, n = 65 severe asthma.

	Healthy control	Non-severe asthma	Severe asthma	p value*
n	42	49	65	
Age	41 ± 13	37 ± 12	44 ± 13	**0.01**
Female, %	60	63	66	0.78
BMI	28 ± 6	30 ± 9	32 ± 8	**0.012**
Race (Non-Hispanic White/African American/Other), %	17/69/14	20/69/10	28/58/14	0.63
Baseline % predicted FEV_1_	99 ± 12	84 ± 15	70 ± 21	**< 0.0001**
Baseline FEV_1_/FVC	0.81 ± 0.04	0.73 ± 0.09	0.69 ± 0.10	**< 0.0001**
FeNO**, ppb	NA	40 ± 41	32 ± 29	0.09
Total serum IgE levels**, IU/ml	72 ± 157	301 ± 455	291 ± 477	**< 0.0001**
Number of positive specific IgE (of 15 tests)	1.57 ± 2.49	5.67 ± 3.82	4.06 ± 4.02	**< 0.0001**
Blood eosinophils**, cells/µl	153 ± 114	276 ± 342	258 ± 251	**0.04**
Sputum % eosinophils**	NA	3.99 ± 8.25	4.58 ± 11.53	0.58

*Kruskal Wallis test for continuous variables or Chi-square test for categorical variables.

**Continuous variables not in normal distribution were presented as median with first and third quartiles.

Significant values are in bold.

Data analysis revealed that airway epithelial cell (AEC) mRNA expression of MYADM was significantly associated with asthma severity (Table 3) and asthma related phenotypes (Table 4). Specifically, increased MYADM expression significantly correlated with asthma and asthma severity in that healthy controls < non-severe asthma < severe asthma (p < 0.0001). In addition, several parameters associated with type 2 high asthma were also significantly associated with MYADM AEC gene expression (Table 4). Most interestingly, in regards to our findings in mouse models and ex vivo eosinophil studies, increased eosinophil counts in the blood significantly correlated with increased MYADM AEC gene expression (p = 0.0044) in asthma patients. While sputum eosinophils showed the same trend, the association did not achieve statistical significance (p = 0.07). Although baseline FEV_1_ (% predicted) was not significantly associated with MYADM expression, there was a slight, yet significant, negative association of MYADM expression to FEV_1_/FVC ratios (p = 0.04) (Table 4). FeNO (p = 0.0007) and the number of exacerbations within the last 12 months (p = 0.0016) were strongly associated with MYADM AEC expression.

**Table 3:** Asthma is associated with MYADM airway epithelial cell gene expression. RNA sequencing (RNA-seq) was performed on airway epithelial cells (AEC) collected from brush biopsies from the Severe Asthma Research Program (SARP) III longitudinal cohort (n = 156). Table reports the p-values from tests of association comparing MYADM AEC mRNA expression between (1) healthy controls and asthmatics and between (2) non-severe asthmatics and severe asthmatics (3) healthy controls, non-severe and severe asthmatics.

	Healthy control (n = 42)	Non-severe asthma (n = 49)	Severe asthma (n = 65)
MYADM mRNA levels*	9.26 ± 0.31	9.50 ± 0.30	9.61 ± 0.32
	Control vs. asthmatics	Non-severe vs. severe	Control vs. non-severe vs. severe
p-value**	**< 0.0001**	0.16	**< 0.0001**

*Natural logarithm transformed.

**A generalized linear model, adjusted for age, sex, BMI, race, and batch effect.

Significant values are in bold.

**Table 4:** Parameters of asthma associated with MYADM airway epithelial cell gene expression. Table reports the correlation coefficient (β) and the corresponding p-values of parameters of asthma associated with MYADM AEC mRNA in 114 subjects with asthma.

Variables relevant to asthma	Correlation coefficient, β	p-value*
Baseline FEV_1_, % predicted	− 8.5	0.12
Baseline FEV_1_/FVC	− 0.06	**0.04**
Blood eosinophil count	0.52	**0.0044**
Sputum eosinophil count, %	0.69	**0.07**
FeNO	0.36	**0.0007**
Total serum IgE level	0.28	0.13
No. of exacerbations in the last 12 months	2.7	**0.0016**

*A generalized linear model, adjusted for age, sex, BMI, race, and batch effect. 114 subjects with asthma were included in the analyses. MYADM mRNA levels were natural logarithm transformed.

Significant values are in bold.

## Discussion

Our previous studies have shown that SP-A has the ability to promote eosinophil apoptosis^20^. However, the receptor(s) through which this occurs was still unclear. Although the Fc receptor appeared to be involved in SP-A binding to eosinophils^16^, it did not appear to modulate all apoptotic events^20^. Therefore, we set out to identify novel proteins on eosinophils that associated with SP-A. Pull-down assays of SP-A and eosinophil lysate followed by LC–MS/MS analysis yielded one transmembrane associated protein, MYADM, and several new intracellular proteins that associated with SP-A: NONO, PRPS1, PRPS2, DNPEP, KPRA and KPRB- which are all intracellular proteins.

NONO is a nuclear protein that is able to recognize specific viral capsids, playing a role in innate immune activation via the cGAS-STING pathway^39^. PRPS1 and PRPS2 together are involved in nucleotide biosynthesis by catalyzing the synthesis of phosphoribosylpyrophosphate (PRPP), whereas KPRA and KPRB bind to PRPS1 and PRPS2, negatively regulating PRPP^35^. DNPEP is a cytosolic aminopeptidase, an enzyme that has preference for aspartate residues^40^, and is important for intracellular metabolism^41^. MYADM, the only membrane-associated^35^ protein pulled-down, is a multi-pass membrane protein that has 8 membrane spanning regions, whose most well-known function is tied to its upregulation during multipotent progenitor cell differentiation, influencing differentiation into myeloid cells^35,36^. Functional experiments revealed that MYADM antibody blockade of this membrane protein significantly diminished the ability of SP-A to induce eosinophil apoptosis in both mouse and human eosinophils in vitro and that inhibition of the SP-A:MYADM interaction resulted in worse asthma phenotypes in two different mouse models.

In the OVA mouse model of experimental allergen-induced inflammation and eosinophilia, disruption of the SP-A:MYADM interaction resulted in persistent eosinophilia and a delay in resolution, likely contributing to the increases in airway hyperreactivity measurements observed. We used HDM as a second mouse model of asthma as it results in a mixed granulocytic inflammation including neutrophils, as well as eosinophils. In this model, we examined differences in lung function independent of a methacholine provocation. We discovered that mice challenged with HDM had a significant increase in total airway resistance (Rrs) and Newtonian resistance (Rn) when MYADM antibody was administered. In fact, the airway responses at day 6 post-terminal challenge of mice that received the antibody were more similar to airway responses previously observed in mice at day 1 post-terminal challenge, suggesting the antibody may have impaired resolution of allergic airways. This finding was accompanied by a delay in mucus clearance as shown by the significantly increased PAS staining in mice that received the antibody at the 6-day time point. We chose to examine these two common models of asthma and ask slightly different but related questions. First, using Ova, we were able to determine the impact of blocking MYADM in eosinophil resolution over the course of 5 days. Second, using HDM, we were able to determine the impact on impaired resolution on non-methacholine provoked airway resistance and mucus persistence in the airways. Taken together, this suggests that disrupting the SP-A:MYADM axis leads to not only an increase and persistence in key phenotypes associated with inflammation (i.e., persistent eosinophilia and mucus), but also a delay in the resolution of constricted airways and airways resistance that is associated with asthma.

In addition to eosinophils, MYADM is expressed in other cell types including epithelial cells in various tissue compartments^42^, and is highly expressed in the brain and lungs^43^. Although little is known about MYADM in regards to lung function, Sun et al. have shown that MYADM promotes extracellular signal-regulated kinase/mitogen-activated protein kinase (ERK/MAPK) dependent proliferation and migration^44^, which results in vascular remodeling in human aortic artery smooth muscle cells. In asthmatics, increased phosphorylated ERK (p-ERK) has also been observed in the airway epithelial cells and smooth muscle cells from endobronchial biopsies of patients with severe asthma, where healthy controls < non-severe asthma < severe asthma in mean fluorescence intensity of staining^45^. In the context of interleukin-13 (IL-13)-induced lung inflammation, a cytokine widely implicated in a variety of asthma phenotypes, the downstream activation of ERK leads to eosinophilic inflammation, mucus production, airway hyperresponsiveness and remodeling^46,47^. Interestingly, while we saw no effect of the blocking antibody in HDM-challenged mice in regards to *Muc5ac*, our saline-treated mice that received the blocking antibody exhibited decreased *Muc5ac* gene expression, which may be associated with decreased p-ERK signaling. Further studies are needed to better define mechanisms by which epithelial-expressed MYADM participates in asthma phenotypes.

MYADM regulation of p-ERK may also play a role in eosinophil viability. While we know that SP-A can induce eosinophil apoptosis, it is also well-established that p-ERK signaling in eosinophils is related to their viability. During resting conditions, ERK is contained within the cytoplasm. However, upon mitogen stimulation, a biphasic activation of ERK1/2 occurs with the initial rapid onset of kinase activity occurring within 5–10 min. The second wave occurs some hours later with another onset of activity that can persist for up to 6 h. Within 15 min of activation, ERK1/2 becomes phosphorylated at threonine and tyrosine residues. The phosphorylation results in the dissociation of ERK1/2 from the cytosolic factors and ERK1/2 translocates to the nucleus. It has been established that ERK1/2 signaling promotes cell survival by two mechanisms: inactivation of the component of the cell death machinery and the increased transcription of pro-survival genes^48^. Clinically, asthmatic subjects had more ERK1/2 activation and mediator release from eosinophils isolated from their airways^49^. In our studies, blockage of the SP-A:MYADM interaction may prevent SP-A from inducing eosinophil apoptosis and/or blocking MYADM may result in decreased p-ERK signaling, which would decrease eosinophil viability. Further studies are needed to help discern the precise mechanisms of action in regards to eosinophil viability.

A notable weakness of our study is that we were unable to associate MYADM expression on human eosinophils with asthma severity, as these samples were not available to us. However, we were able to assess the association of MYADM airway epithelial cell (AEC) mRNA expression with asthma status, along with other parameters of asthma, in a subgroup of the Severe Asthma Research Program (SARP) III cohort. MYADM AEC mRNA expression significantly correlated with asthma severity, where healthy controls < non-severe asthma < severe asthma. Most remarkably, increasing MYADM AEC mRNA expression was also significantly correlated with increased blood eosinophil counts, as well as, decreased FEV_1_/FVC ratio.

While our functional studies with eosinophils show that blocking the SP-A:MYADM interaction resulted in more eosinophilia and worse lung function, the SARP III data suggested increased epithelial expression of MYADM is associated with increased eosinophilia and worse lung function. Little is known about the roles of MYADM in asthma and MYADM may likely have different and complex roles in the many cell types that express it. While we find that SP-A needs MYADM expression on eosinophils in order to properly induce apoptosis, the SARP data suggests increased epithelial MYADM expression is associated with more eosinophils and worse asthma phenotypes. One speculation for this phenomenon could be attributed to previous studies that reported SP-A levels are decreased or dysfunctional in some asthmatic subgroups^19,50^. While we are unable to test this in our current work, future studies should determine if SP-A levels in asthma patients are negatively associated with MYADM levels. Additionally, it will also be important to determine what factors are driving increased MYADM epithelial expression in asthma and the relative expression of MYADM on eosinophils in asthma.

SP-A has been well-described as an innate immune modulator and has been shown to mediate processes relevant to asthma, namely eosinophilic resolution and mucus production. Clinically, obese asthmatics have significantly less SP-A in bronchoalveolar lavage fluid (BALF) as compared to lean asthmatics, which could contribute to enhanced tissue eosinophilia as detected by other research groups^51,52^. While SP-A-based therapies have been suggested, specific receptors and modes of action have not been defined in the asthma field. The clinical implications of this work suggest that in certain individuals with severe asthma, increased expression of MYADM and decreased expression of SP-A, may be a driving factor contributing to their more severe phenotypes. While we have opened to door to many new potentially relevant avenues of study for SP-A, MYADM and asthma, taken together, our studies bring to light a novel binding partner for SP-A that is necessary for SP-A to induce eosinophil apoptosis. Additionally, we show that epithelial expression of MYADM is associated with asthma severity- which warrants further study. While there is much still to be discovered in regards to MYADM and asthma, we believe we are the first to acknowledge the potential importance of this previously unrecognized transmembrane protein in patients with asthma.

## Supplementary Information

Supplementary Figure 1.

Supplementary Legends.
