# Supplementary figures and images for: Myeloid-associated differentiation marker is a novel SP-A-associated transmembrane protein whose expression on airway epithelial cells correlates with asthma severity
Source: Sci Rep. 2021 Dec 3;11:23392. doi: 10.1038/s41598-021-02869-w (PMC8642528; doi:10.1038/s41598-021-02869-w)

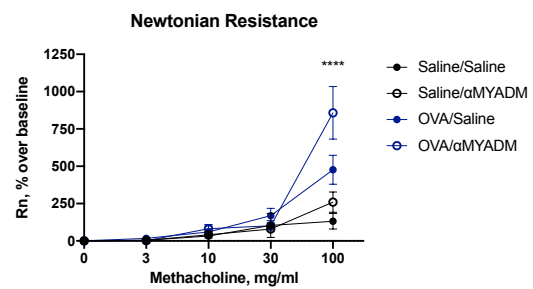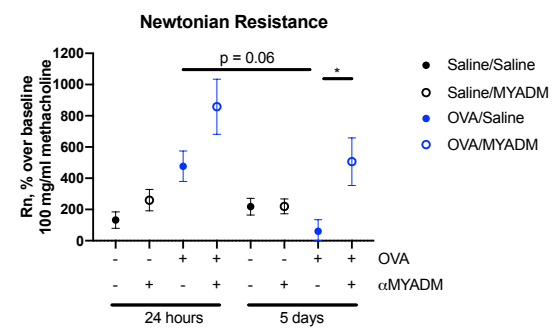

Supplement: Supplementary file 1 — Supplementary Figure 1. [file 41598_2021_2869_MOESM1_ESM.pdf]
