# Supplementary material for: Myeloid-associated differentiation marker is a novel SP-A-associated transmembrane protein whose expression on airway epithelial cells correlates with asthma severity
Source: Sci Rep. 2021 Dec 3;11:23392. doi: 10.1038/s41598-021-02869-w (PMC8642528; doi:10.1038/s41598-021-02869-w)
Supplement: Supplementary file 2 — Supplementary Legends. [file 41598_2021_2869_MOESM2_ESM.docx]

**Supplemental Figure 1. Additional lung function measurements from in vivo models.** Newtonian resistance (R_n_) values as measured by FlexiVent 24 hrs and 5 days after the last OVA challenge. Upper graph is Rn during methacholine challenge 24 hrs post Ova challenge. ****p<0.0001 vs OVA/Saline by multiple t-tests and two-way ANOVA to account for two variables (treatment and methacholine dose). Lower graph is the value of Rn at the administration of 100 μg/ml of methacholine comparing across timepoints, 24 hrs and 5 day values. Multiple t-tests, *p<0.05 by one-way ANOVA for single methacholine dose comparisons. Data (mean + SEM) are from n = 4 – 5 mice per treatment group.
